# Plant growth promotion via priming with volatile organic compounds emitted from *Bacillus vallismortis* strain EXTN-1

**DOI:** 10.3389/fmicb.2024.1524888

**Published:** 2025-01-10

**Authors:** Swarnalee Dutta, Kotnala Balaraju, Soh-Young Oh, Mi-Hyun Lee, Se Weon Lee, Yong Hwan Lee, Kyungseok Park

**Affiliations:** ^1^Division of Biotechnology, Jeonbuk National University, Iksan-si, Republic of Korea; ^2^Crop Protection Division, National Institute of Agricultural Sciences, Wanju-gun, Republic of Korea; ^3^Research Institute of International Agriculture, Technology and Information, Hankyong National University, Anseong-si, Republic of Korea

**Keywords:** *Bacillus vallismortis*, VOCs, heneicosane, induced systemic resistance, seed priming, I-plate assay

## Abstract

Volatile organic compounds (VOCs) produced by potential plant growth-promoting rhizobacteria (PGPR) play an important role in plant interactions. However, the mechanisms underlying this phenomenon are not well understood. Our findings show that the influence of VOCs from the PGPR strain *Bacillus vallismortis* (EXTN-1) on tobacco plant growth is dependent on the culture media used. The VOCs released from sugar-rich media such as potato dextrose agar (PDA) and King’s B (KB) media were highly effective. However, exposure to VOCs from nutrient agar (NA), tryptic soy agar (TSA), and Luria-Bertani (LB) resulted in chlorosis and stunted plant growth. This effect was caused by the discharge of a large amount of ammonia that altered the pH of the plant growth media. Seedlings exposed to VOCs for 10 days exhibited improved growth even after the VOCs were eliminated under greenhouse conditions. Priming of seeds with VOCs for 24 and 48 h induced higher growth than the untreated control, and seeds with 48 h exposure were better as compared to 24 h treatment. Chemical characterization of VOCs emitted by EXTN-1 in different media using solid-phase microextraction (SPME) coupled with gas chromatography-mass spectrometry (GC-MS) showed the presence of 2,3-butanedione and monoxime in all spectra. However, 1-butanol was the prominent peak in VOC of EXTN-1 grown in KB and NA, while acetoin was highest in PDA, followed by KB. Heneicosane and benzaldehyde were exclusively produced in NA media, and these synthetic compounds improved growth in the I-plate assay. This work indicates that VOCs released from EXTN-1 are important for the growth-promoting effect of EXTN-1.

## Introduction

All microorganisms produce volatile organic compounds (VOCs) as part of their normal metabolism (Tilocca et al., 2020; Żuchowska and Filipiak, 2023), and it has been discovered that microbes interact often through the VOCs they release (Tirranen and Gitelson, 2006; Garbeva et al., 2014). The importance of VOCs emitted from the plant growth-promoting rhizobacteria (PGPR) and their contribution to the plant growth promotion and induction of systemic resistance (ISR) to pathogens has already been established (Ryu et al., 2003, 2004). *Bacillus, Pseudomonas, Serratia* and *Stenotrophomonas* are some of the bacterial genera that are known to produce VOCs affecting on the plant growth. Acetoin and 2,3-butanediol from *Bacillus* spp. are identified to enhance the plant growth (Ryu et al., 2003) while the biological functions of several other compounds, including short chain alcohols, aldehydes, acids, esters, ketones, alkanes, alkenes and hydrocarbons are yet to be elucidated (Kai et al., 2009). Such VOCs can act as info chemicals because they occur over a range of concentrations in the biosphere and function over distant spaces (Wheatley, 2002). The VOCs also function as signals, affecting rhizosphere microorganisms and indirectly influencing plant growth (Mhlongo et al., 2022). In addition to improving the plant growth, their ability to elicit induced systemic resistance (ISR) in plants indicates the potential contribution of VOCs toward the effect of PGPR on plants.

The *Bacillus vallismortis* strain EXTN-1 (EXTN-1) was initially isolated from red pepper and has been identified as a potential PGPR for growth promotion and ISR against a variety of pathogens in rice, tobacco, cucumber, tomato and potato (Park et al., 2001, 2006a; 2006b; Park et al., 2007). The ISR was verified by the up-regulation of defense-related gene expression and structural modifications in host plants, as well as the absence of direct antagonism in the antibiosis assay (Park et al., 2001; Ahn et al., 2002). This PGPR strain is capable of eliciting ISR against fungal, bacterial, and viral diseases through salicylic acid (SA), jasmonic acid (JA) and ethylene (ET)-mediated pathways (Park and Kloepper, 2000; Park et al., 2006b). The role of VOCs is yet to be investigated, despite the fact that various aspects of the mechanisms of interaction between EXTN-1 and the host plants have been investigated. The application of PGPR is significantly impeded by the inconsistency that arises under a variety of conditions. Consequently, it is imperative to examine all of the primary mechanisms and determine their appropriate application for agriculture purposes. VOCs are produced by microorganisms, animals, and plants and are distinguished by their high vapor pressure, hydrophobicity, and low molecular weight at ambient temperature. These compounds are capable of performing a variety of vital functions within and across diverse groups of living organisms due to their capacity to traverse porous soils and air without water as a medium (Schulz-Bohm et al., 2018; del Rosario-Cappellari et al., 2019; Suárez-Estrella et al., 2023).

In order to enhance seed germination, seedlings vigor, and to mitigate abiotic stress, a variety of seed priming techniques may be implemented (Nedunchezhiyan et al., 2020; Devika et al., 2021). In addition to these advantages, the biopriming method offers the additional benefit of managing biotic stress. The term “biopriming” typically denotes the utilization of beneficial microorganisms, in particular plant growth-promoting bacteria (PGPB), which are capable of surviving under a variety of challenging environmental conditions (Fiodor et al., 2023). A previous study demonstrated that the use of microbial inoculants containing plant growth-promoting bacteria (PGPB) can increase crop production by 12–20% (Pérez-Jaramillo et al., 2016). Additionally, the biopriming of seeds with a variety of beneficial bacteria, particularly PGPB, can enhance the plant’s resilience and effectiveness in adverse conditions (Chakraborti et al., 2021). The concept of priming plants with PGPR to promote growth and induce eventual defense responses on pathogen attack has been an interesting topic of research (Kandasamy et al., 2009).

The majority of VOCs investigations are conducted under controlled conditions, with a recent shift in focus to field applications. However, several parameters, including soil nutrient status, must be analyzed to produce consistent results in the field environment. Therefore, the current investigation was conducted to evaluate the significance of bacterial culture media and the impact of VOCs emitted by EXTN-1 in the I-plate assay. We also endeavored to investigate the impact of priming seeds with VOC on growth promotion under greenhouse conditions, as the mode of application of VOC for growth enhancement is a critical factor in commercial success. The primary compounds responsible for the effect on plant growth were identified through chemical analysis of VOCs from EXTN-1 in various media. Additionally, some lesser-known VOCs were identified as potential plant growth promoters.

## Materials and methods

### Bacterial strain and selection of suitable culture media

The bacterial strain *Bacillus vallismortis* EXTN-1 was used in all the experiments. Stock cultures were maintained in tryptic soy broth (TSB, Difco, MI, USA) containing 20% sterilized glycerol at −80°C. To assess the effect of culture media on the production of VOCs in the I-plate assay, we selected several commonly used media for PGPR. These media include tryptic soy agar (TSA), which is rich in proteins, nutrient agar (NA), which offers a wide range of nutrients, and Luria-Bertani agar (LB), which is a non-selective, complex, and frequently used culture medium for bacterial growth in laboratories. King’s B agar (KB), which are primarily used for *Pseudomonas* species, and sugar-enriched potato dextrose agar (PDA) were also used as media that were non-specific to *Bacillus* strains. For comparative analysis, water agar was used as a control. The initial pH of all the media was approximately 7.

### Plant growth promotion by EXTN-1 *in vitro* using the I-plate assay

The I-plate assay was conducted using a model plant tobacco (*Nicotiana tabacum* cv. Xanthi-nc), which is an optimal choice for the analysis of fundamental biological functions, including plant-microbe interaction, growth responses and environmental responses. Seeds were surface-disinfected using the method described by Park et al. (2013). In brief, the seeds were first treated with a 1% sodium hypochlorite solution, then immersed in 70% ethanol for 3 min, and finally rinsed in sterilized distilled water (SDW). The seeds were then dried on a clean bench and placed on one side of an I-plate containing half-strength Murashige and Skoog (MS) agar medium with pH 5.7 (Duchefa Biochemie B.V., The Netherlands), five seeds per plate. Bacterial cell suspensions of EXTN-1 (20 μl) at 10^8^ cfu/ml were impregnated in a sterile paper disk and placed on the other side of the I-plate containing different culture media, as described before. The plates were completely sealed with parafilm and incubated for 10 days at 25°C with a photoperiod of 12 h light and 12 h darkness. The growth of plants was measured as fresh weight. A similar experiment was conducted to remove ammonia, with 5 ml of 0.74 mM phosphoric acid placed in a third chamber established in I-plates to trap the ammonia emitted from EXTN-1 growing in various media (Weise et al., 2012). The production of ammonia by EXTN-1 in various media was determined using Nessler’s reagent (K_2_HgI_4_, which is prepared by mixing 2 g potassium iodide in 5 ml of SDW. To this solution, 3 g of mercury (II) iodide is added, and the volume is made to 20 ml. Finally, 40 g potassium hydroxide (30%) is added to form the alkaline base).

### Seed priming with VOCs on growth of plants under greenhouse condition

To determine the effect of seed priming with VOCs emitted by EXTN-1, surface-disinfected tobacco seeds were placed on half-strength MS media in an I-plate. Initially seeds were primed for 4, 8, 12, 24, and 48 h, with VOCs from EXTN-1 grown in various media, as described above. However, there was no difference in germination or growth when seeds were treated for ≤12 h. Therefore, seeds primed for 24 h and 48 h were further used as priming period for this study. The EXTN-1 culture plates were removed after the appropriate time period, and seeds were allowed to germinate. After 1 week of growth, the germinated seedlings were transferred to soilless potting mixture (TKS2, Flora Gard Ltd., Germany) under greenhouse conditions (25°C, 60–70% humidity and 12 h/12 h light and dark condition) and monitored for growth improvement. Fresh and dry weights of plants were recorded after 4 weeks of growth.

### Identification of VOCs released from EXTN-1

EXTN-1 was cultured in different media, as described above, in 20 ml glass headspace bottles (Supelco, Bellefonte, PA, USA) at 28°C for 24 h. The VOCs were collected and analyzed using solid-phase micro-extraction (SPME) and gas chromatography-mass spectrophotometry (GC-MS) (Farag et al., 2006) with minor modifications (Park et al., 2015). In brief, vials containing EXTN-1 VOCs were injected with SPME fiber and then incubated at 50°C for 30 min. A Bruker 320 mass spectrophotometer and a Bruker 450-GC gas chromatograph were used to conduct the GC-MS analysis. The following program was used to run the SPME fiber: desorption for 2 min at 60°C, commencing with a column temperature of 35°C for 3 min. The temperature was then increased to 180°C at a rate of 10°C/min, then further increased to 240°C at 4°C/min, and was held for 5 min. Helium gas was used as a carrier for the GC-MS, which was run for 37 min at a flow rate of 1 ml/min. The MS was operated at 70 eV in electron ionization mode with continuous scanning from 50 m/z to 500 m/z at a temperature source of 220°C. The NIST/EPA/NIH Mass Spectral Libraries were used to investigate the mass spectra of the volatile compounds.

### Confirmation of growth promotion by selected potentially unreported synthetic VOCs

Although acetoin and 2,3-butanediol were identified as major VOCs produced by EXTN-1, we identified previously lesser-known VOC compounds by GC-MS analysis from EXTN-1 strain when grown in NA media. These lesser-known VOCs were tested for their ability to promote growth of tobacco seedlings *in vitro* using I-plate assay. We tested three synthetic VOCs at different concentrations in an I-plate assay, as described above. Surface-sterilized tobacco seeds were placed on one side of I-plate containing half-strength MS medium. Synthetic compounds of dimethyl disulfide, benzaldehyde, and heneicosane from Sigma Chemicals (Sigma Aldrich, CA, USA) were dissolved in methanol at three different concentrations (5, 50, and 500 ng) and 30 μl was embedded in sterilized paper disks, and were placed in one side of the I-plate. Methanol served as the control. Plates were sealed and incubated at 25°C under a 12 h light/12 h dark photoperiod for 2 weeks and fresh weight of plants was measured.

### Statistical analysis

The data were subjected to analysis of variance using SAS JMP software (SAS Institute, Cary, NC, USA). Significant differences in the treatment means of each sample were determined using the Least Significance Difference (LSD) Test at *P* = 0.05. All experiments were performed at least twice with five replicates each. The data from each experiment were analyzed separately.

## Results

### Effect of VOCs derived from the strain EXTN-1 on plant growth promotion *in vitro*

**FIGURE 1:**
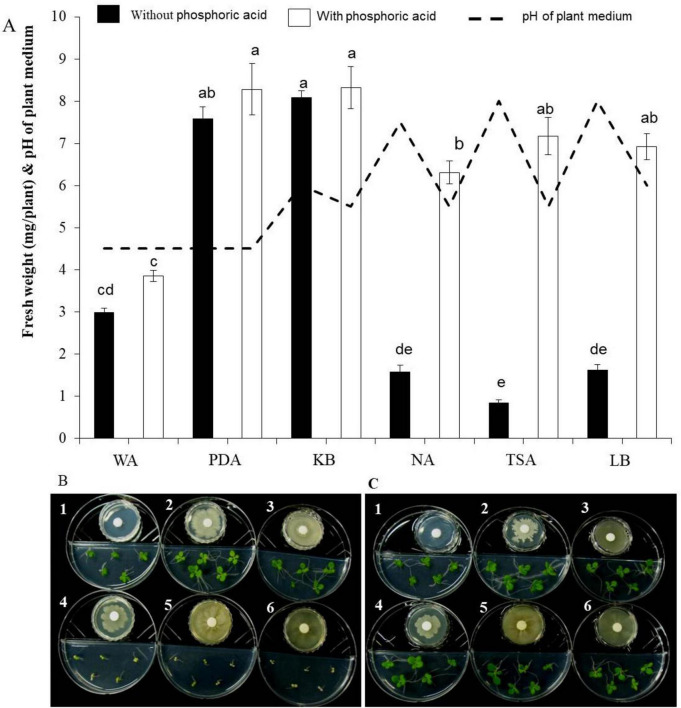
Effect of VOCs from EXTN-1 grown in different media on tobacco growth. (1) water agar (WA) (2) potato dextrose agar (PDA) (3) Kings’ B (KB) (4) nutrient agar (NA) (5) tryptic soy agar (TSA) (6) and Luria-Bertani agar (LB). **(A)** Fresh weight of plant and pH of medium 10 days after the growth. The experiment was repeated at least once. Bars with the same letters do not differ from each other according the least significant difference (LSD) (*P* = 0.05). **(B)** Growth of plants in I-plate assay and **(C)** Assay with phosphoric acid in I-plate to trap ammonia.

**FIGURE 2:**
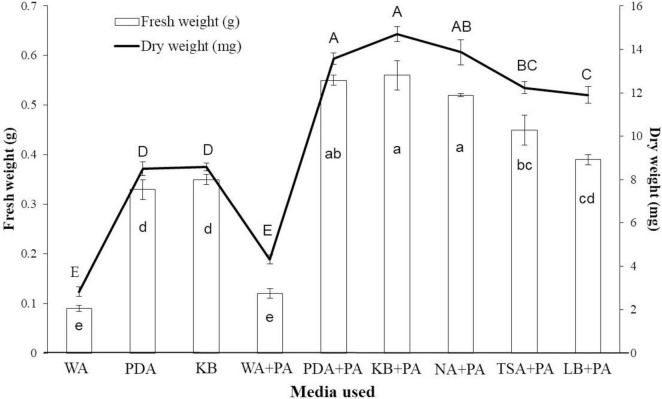
Effect of initial exposure of VOCs from EXTN-1 grown in different media on tobacco plants when transferred to greenhouse condition. Seedlings exposed to VOCs in I-plate assay were transferred to greenhouse conditions. Fresh and dry weight of tobacco seedlings was measured 4 weeks after they were removed from the VOC emitted from the EXTN-1 cultured in various media, including WA, PDA, KB, WA + phosphoric acid (PA), PDA + PA, KB + PA, NA + PA, TSA + PA, and LB + PA. Data presents mean ± SD and bars with the same letters do not differ from each other according to the least significant difference (LSD) (*P* = 0.05).

When tobacco seeds were exposed to VOCs emitted by the EXTN-1 strain during its culture for 4 weeks in I-plates, plant growth improved in terms of fresh weight (Figure 1A). The increase was 3-fold and 4-fold for VOCs from PDA and KB media, respectively. When the EXTN-1 strain was cultured in PDA or KB media, the VOCs promoted plant growth; but, when grown in NA, TSA or LB media, the VOCs released from EXTN-1 had a detrimental effect on plants, resulting in stunted growth and severe chlorosis (Figure 1B). The pH of the media increased to an alkaline level for NA (7.5), TSA (8.0), and LB (8.0). However, when cultured in the presence of 0.74 mM phosphoric acid, the negative effects of VOCs from these media were not observed (Figure 1C), and VOCs from EXTN-1 in all media considerably improved plant growth (Figure 1A). All the medium maintained a pH between 4.5 to 6. The ammonia production for EXTN-1 in different media was the highest in TSA (6-fold) and LB (5-fold), while with NA and KB showing a 3-fold increase. The lowest ammonia level was recorded in PDA with a one-fold increase compared to the WA control (Table 1). Even after the VOC was removed, tests in greenhouse and *in vitro* confirmed that it had positive influence on the growth of tobacco seedlings. The growth pattern of seedlings transferred to greenhouse conditions was found to be identical to that in I-plates. Compared to seedlings treated with VOCs from WA, seedlings grown in PDA and KB exposed to VOCs released from EXTN-1 had a growth rate in fresh weight that was more than 3-fold higher after 4 weeks (Figure 2). Fresh weight was the highest in KB, PDA and NA after ammonia was removed. The dry weight of plants that were exposed to VOCs from KB, PDA, and NA was approximately three times greater than that of plant that were exposed to VOCs from WA (Figure 2).

**TABLE 1:** Estimation of ammonia emitted by EXTN-1 in different media.

Media	Ammonia production (mg/L culture)
WA	5.279 ± 0.01^c^
PDA	5.326 ± 0.01^c^
KB	20.116 ± 0.07^b^
NA	20.814 ± 0.16^b^
TSA	32.326 ± 0.07^a^
LB	31.093 ± 0.11^a^

Data represent mean + standard error. The figures followed by different letter indicate significant differences among the treatments at *P* = 0.05.

### Impact of seed priming with VOC on growth of plants under greenhouse condition

**FIGURE 3:**
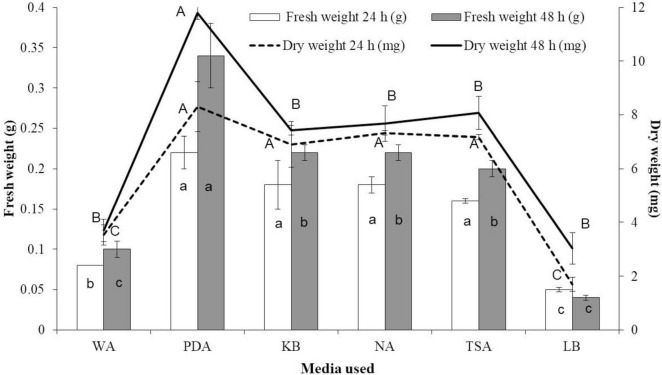
Effect of seed priming with VOCs from EXTN-1 grown in different media under greenhouse conditions. The fresh and dried weights of plants were recorded 4 weeks after the growth. Data presents mean ± SD and bars with the same letters do not differ from each other according to the least significant difference (LSD) (*P* = 0.05). The media used were water agar (WA), potato dextrose agar (PDA), Kings’ B (KB), nutrient agar (NA), tryptic soy agar (TSA), and Luria-Bertani agar (LB).

Seeds primed with VOC derived from EXTN-1 in different media for ≤12 h did not show any difference in germination or growth as compared to the control. Seeds primed for 24 and 48 h showed growth improvement with faster germination when grown in MS media for 1 week. Then the seedlings were transferred to greenhouse in pots, where they displayed consistent enhanced growth (Figure 3). There was no difference in the germination percentage among the seeds with different treatments. But comparatively, plants from 48-h-exposed seeds were found to be healthier than seedlings with 24-h-VOC-treated seeds in terms of biomass. After 4 weeks of growth in greenhouse, fresh and dry weights were recorded highest with more than 3-fold increase in seeds exposed to VOCs from EXTN-1 grown in PDA for 48 h followed by KB, NA, and TSA (Figure 3). The growth of plants exposed to VOCs emitted from EXTN-1 cultured on LB was low as compared to control and other treatments.

### Chemical profile of VOCs from EXTN-1 using SPME and effect on plant growth

**FIGURE 4:**
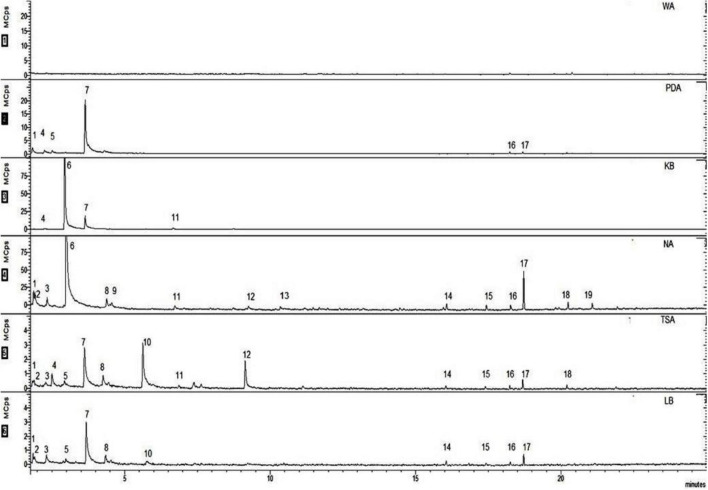
Identification of VOCs emitted by EXTN-1 in different media in headspace flasks at 28°C for 24 h. The SPME-GC-MS system was employed to conduct the analysis. The numbers represent the volatiles as follows: (1) carbon dioxide (2) 1,4-pentadiene (3) 2-butanone (4) oxybis-dichloromethane (5) 2,3-butanedione (6) 1-butanol (7) acetoin (8) 1-pentanol (9) dimethyl disulfide (10) 2,3-butanediol (11) acetic acid, butyl ester (12) 2,5-dimethyl pyrazine (13) benzaldehyde (14) hexadecane (15) tetradecane (16) non-amethyl-3-(trimethylsiloxy)-tetrasiloxane (17) 4-methyl tridecane (18) tetramethyl silane (19) heneicosane.

A chemical profile of the VOCs emitted by EXTN-1 in various media was obtained using SPME coupled with GC-MS. Depending on the culture media, the spectrum of VOCs varied. The molecular weights of all of these compounds ranged from 44 to 296 and their retention times were between 2 and 25 min (Figure 4). The VOC 2,3-butanedione, monoxime was detected in PDA and in trace amounts in KB-grown EXTN-1 VOCs. Acetoin was the most prominent peak in the spectra of VOCs from EXTN-1 grown in all media except NA. The peak for 1-butanol was the highest in NA, followed by KB. Carbon dioxide was emitted by EXTN-1 in NA, with traces of the gas in PDA, TSA, and LB. However, the peak was not detected in the VOC profile of EXTN-1 grown in KB. Hexadecane, tetradecane, and tridecane were the most frequently detected long-chain VOCs in the NA, TSA, and LB media that were used to culture EXTN-1. Despite the fact that the pattern of peaks was comparable in NA, TSA, and KB, the quantity of emitted VOCs was significantly higher in NA (Figure 4). The peak 2,3-butanediol was observed in EXTN-1 that was cultured in TSA and LB. Dimethyl disulfide, benzaldehyde, and heneicosane were the only peaks that were exclusively evident in the spectrum of NA.

**FIGURE 5:**
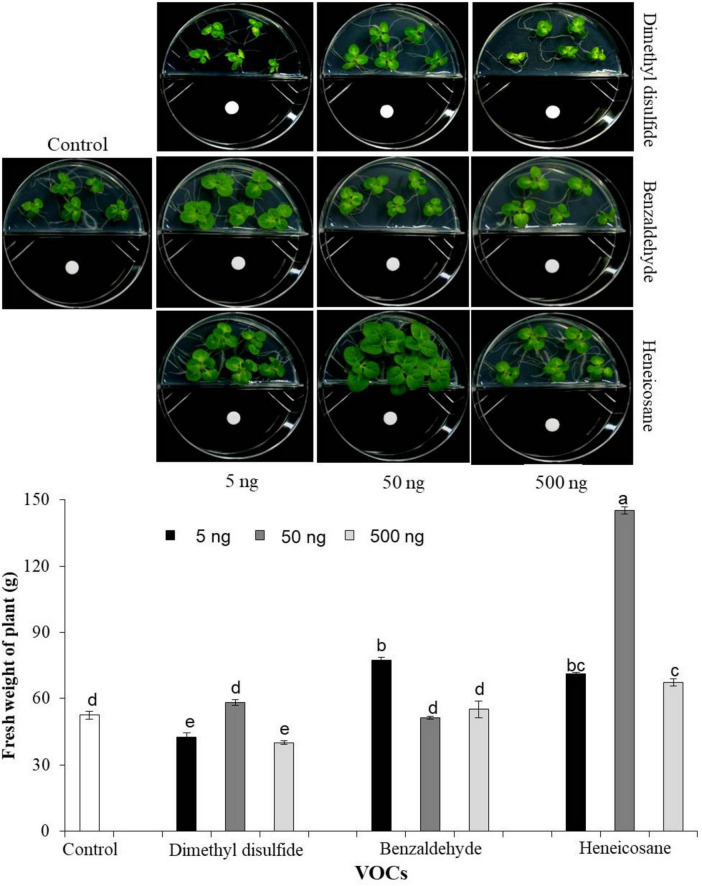
Effect of three different concentrations (5, 50, and 500 ng) of synthetic VOCs identified from EXTN-1. Fresh weight of plants was determined by embedding synthetic compounds of dimethyl disulfide, benzaldehyde and heneicosane dissolved in methanol were embedded in sterile paper disks and placed onto one side of the I-plate. Surface sterilized tobacco seeds were placed on the other half of the plate, and growth was assessed 2 weeks after the growth in comparison with the control (methanol). Data presents mean ± SD and bars with the same letters do not differ from each other according to the least significant difference (LSD) (*P* = 0.05).

Further analysis by application of these three synthetic VOCs on tobacco enhanced growth. Although lower concentrations of benzaldehyde improved growth (Figure 5), the growth enhancement was not consistent with increase in concentration of either benzaldehyde or dimethyl disulfide. However, the growth of tobacco plants was significantly augmented in comparison to the control group when exposed to the synthetic compound heneicosane at three different concentrations (Figure 5). The fresh weight of plants increased by 3-fold in the presence of heneicosane (50 ng). The plant fresh weight increased significantly with a lower and higher concentration of heneicosane (5 and 500 ng) as compared to control, but the optimal result was achieved with a moderate concentration of 50 ng (Figure 5). Plant growth was enhanced by dimethyl disulfide treatment at 50 ng; however, the fresh weight of the plant was low at 5 and 500 ng in comparison to the control in the *in vitro* assay (Figure 5).

## Discussion

Previous research has explored the significance of VOCs in the context of ISR and plant growth (Ryu et al., 2004; Tahir et al., 2017). Differential VOC emission from rhizosphere bacteria has the potential to influence the architecture of the root system and promote plant growth (Almeida et al., 2023). Our research suggests that VOCs are essential for the beneficial impact of EXTN-1 on plants; however, the impact on plant growth is contingent upon the bacterial culture media. It has been reported that the number and spectrum of volatiles produced by bacteria are dependent upon the availability of nutrients and the growth conditions (Weise et al., 2012; Garbeva et al., 2014; Rani et al., 2023). In sugar-enriched media, the VOCs from EXTN-1 promoted plant growth, whereas in nitrogenous media, VOCs inhibited plant growth by causing leaf chlorosis. This is a consequence of the alkalinization of the plant medium by ammonia. Previous research has demonstrated that ammonia is a significant bioactive compound in bacterial volatiles, which is responsible for significant growth inhibition of plants (Weise et al., 2013). It has been reported that a rapid influx of NH_3_ into plant cells occurred when the external pH was high. This phenomenon may result in a variety of ammonia-induced changes in the cells, such as reduced growth and chlorosis (Plieth et al., 2000; Liu and von Wirén, 2017). Soil pH changes can have a significant impact on iron uptake by hosts since niche pH affects this process (Zhang et al., 2009). Bacterial VOCs may play a pivotal role in dictating the dynamics of rhizosphere microbial community; given that soil pH is critical for microbiome composition (Lakshmanan et al., 2014). A recent study found that the growth of plants was indirectly influenced by the alteration of the rhizosphere microbiome by PGPR and VOCs such as indole (Yaghoubi Khanghahi et al., 2024). However, the negative effect was not observed when the ammonia was confined with phosphoric acid. Conversely, in the absence of ammonia, VOCs from EXTN-1 had a beneficial effect on exposed plants, irrespective of the culture media. This is an important aspect of the investigation, as the fundamental experiment utilizes the I-plate assay to evaluate the effect of VOCs from PGPR on plant growth. The outcome may be either positive or negative, as the growth media plays a significant role in the VOCs emitted by bacteria. This may result in the elimination of certain potential bacterial sources of VOC based on an experiment that utilizes only one media. In certain instances, the impact of beneficial VOCs may be obscured by other volatiles, such as ammonia in this case. The potency of bacterial volatiles necessitates a comprehensive investigation. In this study we have selected some of the most frequently used media for PGPR strains in the laboratory, particularly in the I-plate assay to identify VOCs such as TSA, NA and LB. Additionally, we have included media such as PDA, which is used for antifungal analysis. Because VOCs differ between culture media, they may alter laboratory assays used to characterize the PGPR strain. Furthermore, soil nutrient level may alter the production of VOCs, determining the ultimate outcome of PGPR strain application under field conditions.

When seedlings were transferred to greenhouse after 10 days of exposure, their growth consistently increased compared to the control group. The results of this study show that initial exposure is an important and sufficient to carry on the positive effect. When VOCs were used to prime seeds for 24 and 48 h, the result was the same with faster germination of seeds as compared to the control. When it came to fresh and dry weight, seedlings that were exposed for 48 h did better than seeds that were exposed for 24 h. When EXTN-1 was grown in NA and TSA, the negative effect observed in I-plates in seedlings exposed to VOCs was not observed in seed-primed plants under greenhouse conditions. There was a statistically significant increase in growth, in comparison to the control group. The leaves were in good health and did not exhibit any symptoms of chlorosis. Our research indicates that the growth of plants is facilitated by the short-term priming of them with VOCs, despite the fact that additional research is required to confirm this effect. This outcome is crucial for the practical implementation of VOCs in field conditions. Seed-priming with live cells of PGPR is a well-established phenomenon. It has also been reported that the treatment of roots with suspensions of VOC from PGPR results in induced resistance against biotic stress (Farag et al., 2013). However, this is the initial report of seed priming with VOCs, to the best of our knowledge. Further analysis of seed exposure at various time points, as well as the concentration of VOCs required for priming, will identify the threshold level.

The chemical profile of VOCs from EXTN-1 demonstrated the emission of several bioactive compounds, including previously reported growth-modulating VOCs such as acetoin, 1-butanol, and 2,3-butanedione (Ryu et al., 2003, 2004; Scala et al., 2013). Acetoin was the most prominent peak in all media (excluding NA), and it can be inferred that it is the growth-promoting VOC from EXTN-1 in these media. The growth improvement in plants was significant in KB plates, despite the fact that EXTN-1 produced a similar quantity of ammonia in KB and NA media. Conversely, the growth in NA plates was reduced. One potential explanation is the duration of time required to alkalize the medium. The pH of the plant medium in KB increases to 6 over an 8-day period when exposed to EXTN-1. However, in NA, the pH reached 7 on the 3rd day (detailed data not shown). The pH increases abruptly on the 2nd day for TSA and LB. Normal growth is not impeded in KB plates, as the plants are provided with a suitable medium for growth for a period of 8 days, Acetoin contributes to the growth improvement.

The peak for acetoin was not detected in the VOCs of EXTN-1 grown in NA. However, upon exposure to plants, the VOCs increased plant growth in the I-plate assay once the ammonia was contained (Figure 1). The primary peak in NA and KB media has been identified as 1-butanol. Acetoin and 2,3-butanediol were not detected in NA media, so we tested some of the other compounds identified in the spectrum to isolate the VOC related to growth promotion. The peaks were highest in NA and TSA media, with the exception of benzaldehyde, dimethyl sulfide and heneicosane that were exclusively detected when EXTN-1 was grown in NA medium. Benzaldehyde has been identified as a microbial VOC that inhibits plant growth (Kanchiswamy et al., 2015). *Arabidopsis* was differentially affected by the emission of benzaldehyde by lemon rhizobacteria (Guitierrez-Luna et al., 2010). Therefore, further in-depth analysis of VOC concentration is required. Dimethyl disulfide has been identified as a catabolic product of the sulfur-containing amino acids methionine and cysteine. It has been reported to promote growth in *Nicotiana attenuata* by enhancing sulfur nutrition (Meldau et al., 2013), the same effect is not observed in our experiment. With the exception of the 50 ng concentration, which only slightly increased fresh weight of tobacco in comparison to the control, the other concentrations did not have positive impact on the plant growth.

Other research has suggested that heneicosane may be a contributing factor to the antimicrobial properties of the sea urchin *Temnopleurus alexandri* and the medicinal plant *Pulicaria crispa* (Elshiekh and Mona, 2015). Currently, there is no report available regarding the possible enhancement of plant growth by heneicosane as a VOC from PGPR. Our *in vitro* findings imply that heneicosane is a promising VOC that promotes tobacco growth. The issue would be to identify the concentration needed to provide a similar growth-promoting effect across diverse plant species and under field conditions. Several long-chain decanes were detected in the VOCs of EXTN-1, including hexadecane, tertadecane, and tridecane. Hexadecane and tridecane from *Paenibacillus polymyxa* were reported earlier to be responsible for ISR in *Arabidopsis* against *Pectobacterium carotovorum* and *Pseudomonas syringae* (Lee et al., 2012; Park et al., 2013). The positive function of long-chain decanes is suggested by the fact that tetradecane increased the fresh weight of plants at low concentrations in this study. The results of this investigation suggested that the growth of plants was influenced by the concentration of VOC that was administered. The growth promoting effect is dose-dependent, and the quantity of VOC produced by PGPR is a determining factor in the resultant effect of plants, as indicated by a similar earlier report (Bailly and Weisskopf, 2012; Bitas et al., 2013).

In conclusion, our research demonstrates the significance of VOCs emitted by EXTN-1 in the promotion of tobacco growth through the short-term priming of seeds or seedlings, which can be exploited for field application. It has been observed that the majority of the VOCs released by EXTN-1, including acetoin, carbon dioxide, long-chain decanes, benzaldehyde, and dimethyl disulfide, have the ability to promote plant growth in various conditions. Another important factor in deciding the effect of on plant growth is the concentration of VOC. With the identification of heneicosane as one of the promising VOCs that contribute plant growth, further analysis is needed to optimize the concentration and conditions required. Consequently, the results obtained under controlled conditions may not precisely reflect the actual picture of the impact of VOCs from PGPR in the field. However, factors such as the influence on competing microbial communities and the impact on the environment must be thoroughly investigated prior to practical application in the field. Further research is required in several areas, including the optimal concentration of VOCs, effects on plant physiology, and consistency in different crops. Nevertheless, the present results can be taken as basic analysis for further detailed experiments that are essential for understanding the diverse factors involved in the mechanisms during the interaction of PGPR and/or VOCs with host plants.

## Data Availability

The raw data supporting the conclusions of this article will be made available by the authors, without undue reservation.

## References

[B1] Ahn I. P. Park K. S. Kim C. H. (2002). Rhizobacteria-induced resistance perturbs viral disease progress and triggers defense-related gene expression. *Mol. Cells* 13 302–308. 12019515

[B2] Almeida O. A. C. de Araujo N. O. Mulato A. T. N. Persinoti G. F. Sforça M. L. Calderan-Rodrigues M. J. (2023). Bacterial volatile organic compounds (VOCs) promote growth and induce metabolic changes in rice. *Front. Plant Sci.* 13:1056082. 10.3389/fpls.2022.1056082 36844905 PMC9948655

[B3] Bailly A. Weisskopf L. (2012). The modulating effect of bacterial volatiles of plant growth current knowledge and future challenges. *Plant Signal. Behav.* 7 79–85. 22301973 10.4161/psb.7.1.18418 PMC3357376

[B4] Bitas V. Kim H.-S. Bennett J. W. Kang S. (2013). Sniffing on microbes: diverse roles of microbial volatile organic compounds in plant health. *Mol. Plant Microbe Interact.* 26 835–843. 23581824 10.1094/MPMI-10-12-0249-CR

[B5] Chakraborti S. Bera K. Sadhukhan S. Dutta P. (2021). Bio-priming of seeds: plant stress management and its underlying cellular, biochemical and molecular mechanisms. *Plant Stress* 3:100052.

[B6] del Rosario-Cappellari L. Chiappero J. Banchio E. (2019). Invisible signals from the underground: a practical method to investigate the effect of microbial volatile organic compounds emitted by rhizobacteria on plant growth. *Biochem. Mol. Biol. Educ*. 47 388–393. 30964236 10.1002/bmb.21243

[B7] Devika O. S. Singh S. Sarkar D. Barnwal P. Suman J. Rakshit A. (2021). Seed priming: a potential supplement in integrated resource management under fragile intensive ecosystems. *Front. Sustain. Food Syst*. 5:654001. 10.3389/fsufs.2021.654001

[B8] Elshiekh Y. H. Mona A. (2015). Gas chromatography-mass spectrometry analysis of *Pulicaria crispa* (whole plant) petroleum ether extracts. *Am. J. Res. Commun.* 3 58–67.

[B9] Farag M. A. Ryu C. M. Sumner L. W. Pare P. W. (2006). GC-MS SPME profiling of rhizobacterial volatiles reveals prospective inducers of growth promotion and induced systemic resistance in plants. *Phytochemistry* 67 2262–2268. 16949113 10.1016/j.phytochem.2006.07.021

[B10] Farag M. A. Zhang H. Ryu C. M. (2013). Dynamic chemical communication between plants and bacteria through airborne signals: induced resistance by bacterial volatiles. *J. Chem. Ecol.* 39 1007–1018. 23881442 10.1007/s10886-013-0317-9 PMC3738840

[B11] Fiodor A. Ajijah N. Dziewit L. Pranaw K. (2023). Biopriming of seed with plant growth-promoting bacteria for improved germination and seedling growth. *Front. Microbiol*. 14:1142966. 10.3389/fmicb.2023.1142966 36925481 PMC10011460

[B12] Garbeva P. Hordijk C. Gerards S. de Boer W. (2014). Volatiles produced by mycophagous soil bacterium *Collimonas*. *FEMS Microbiol. Ecol.* 87 639–649. 24329759 10.1111/1574-6941.12252

[B13] Guitierrez-Luna F. M. Lopez-Bucio J. Altamirano-Hernandez J. Valencia-Cantero E. Cruz H. R. Macias-Rodriguez L. (2010). Plant growth-promoting rhizobacteria modulate root-system architecture in *Arabidopsis thaliana* through volatile organic compound emission. *Symbiosis* 51 75–83.

[B14] Kai M. Haustein M. Molina F. Petri A. Scholz B. Piechulla B. (2009). Bacterial volatiles and their action potential. *Appl. Microbiol. Biotechnol.* 81 1001–1012. 19020812 10.1007/s00253-008-1760-3

[B15] Kanchiswamy C. N. Malnoy M. Maffel M. E. (2015). Chemical diversity of microbial volatiles and their potential for plant growth and productivity. *Front. Plant Sci.* 6:151. 10.3389/fpls.2015.00151 25821453 PMC4358370

[B16] Kandasamy S. Loganathan K. Muthuraj R. Duraisamy S. Seetharaman S. Thiruvengadam R. (2009). Understanding the molecular basis of plant growth promotional effect of *Pseudomonas fluorescens* on rice through protein profiling. *Proteome Sci.* 7:47. 10.1186/1477-5956-7-47 PMC2805620 20034395

[B17] Lakshmanan V. Selvaraj G. Bais H. P. (2014). Functional soil microbiome: below-ground solutions to an aboveground problem. *Plant Physiol.* 166 689–700. 25059708 10.1104/pp.114.245811 PMC4213098

[B18] Lee B. Farag M. A. Park H. B. Kloepper J. W. Lee S. H. Ryu C. M. (2012). Induced resistance by a long-chain bacterial volatile: elicitation of plant systemic defense by a C13 volatile produced by *Paenibacillus polymyxa*. *PLoS One* 7:e48744. 10.1371/journal.pone.0048744 23209558 PMC3509098

[B19] Liu Y. von Wirén N. (2017). Ammonium as a signal for physiological and morphological responses in plants. *J. Exp. Bot*. 68 2581–2592. 28369490 10.1093/jxb/erx086

[B20] Meldau D. G. Meldau S. Hoang L. H. Underberg S. Wunsche H. Baldwin I. T. (2013). Dimethyl disulfide produced by the naturally associated bacterium *Bacillus* sp B55 promotes *Nicotiana attenuata* growth by enhancing sulfur nutrition. *Plant Cell* 25 2731–2747. 23903320 10.1105/tpc.113.114744 PMC3753394

[B21] Mhlongo M. I. Piater L. A. Dubery I. A. (2022). Profiling of volatile organic compounds from four plant growth promoting rhizobacteria by SPME-GC-MS: a metabolomics study. *Metabolites* 12:763. 10.3390/metabo12080763 PMC9414699 36005635

[B22] Nedunchezhiyan V. Velusamy M. Subburamu K. (2020). Seed priming to mitigate the impact of elevated carbon dioxide associated temperature stress on germination in rice (*Oryza sativa* L.). *Arch. Agron. Soil Sci.* 66 83–95.

[B23] Park H. B. Lee B. Kloepper J. W. Ryu C. M. (2013). One shot-two pathogens blocked: exposure of *Arabidopsis* to hexadecane, a long chain volatile organic compound, confers induced resistance against both *Pectobacterium carotovorum* and *Pseudomonas syringae*. *Plant Signal. Behav.* 8: e24619. 23603940 10.4161/psb.24619 PMC3906419

[B24] Park K. S. Ahn I. P. Kim C. H. (2001). Systemic resistance and expression of the pathogenesis-related genes mediated by the plant growth-promoting rhizobacterium *Bacillus amyloliquefaciens* EXTN-1 against anthracnose disease in cucumber. *Mycobiology* 29 48–53.

[B25] Park K. S. Kloepper J. W. (2000). Activation of PR1a promoter by rhizobacteria that induce systemic resistance in tobacco against *Pseudomonas syringae* pv. *tabaci*. *Biol. Cont.* 18 2–9.

[B26] Park K. S. Paul D. Yeh W. H. (2006a). *Bacillus vallismortis* strain EXTN-1 mediated growth promotion and disease suppression in rice (*Oryza sativa* L.). *Plant Pathol. J.* 22 278–282.

[B27] Park K. S. Paul D. Kim Y. K. Nam K. W. Lee Y. K. Choi H. W. (2007). Induced systemic resistance by *Bacillus vallismortis* EXTN-1 suppressed bacterial wilt in tomato caused by *Ralstonia solanacearum*. *Plant Pathol. J.* 23 22–25.

[B28] Park K. S. Paul D. Ryu K. R. Kim E. Y. Kim Y. K. (2006b). *Bacillus vallismortis* strain EXTN-1 mediated systemic resistance against potato virus X and Y (PVX and PVY) in the field. *Plant Pathol. J.* 22 360–363.

[B29] Park Y. S. Dutta S. Ann M. Raaijmakers J. Park K. (2015). Promotion of plant growth by *Pseudomonas fluorescens* strain SS101 *via* novel volatile organic compounds. *Biochem. Biophys. Res. Commun.* 461 361–365. 25892516 10.1016/j.bbrc.2015.04.039

[B30] Pérez-Jaramillo J. E. Mendes R. Raaijmakers J. M. (2016). Impact of plant domestication on rhizosphere microbiome assembly and functions. *Plant Mol. Biol*. 90 635–644. 26085172 10.1007/s11103-015-0337-7 PMC4819786

[B31] Plieth C. Sattelmacher B. Knight M. R. (2000). Ammonium uptake and cellularalkalization in roots of *Arabidopsis thaliana*: the involvement of cytoplasmic calcium. *Physiol. Plant.* 110 518–523.

[B32] Rani A. Rana A. Dhaka R. K. Singh A. P. Chahar M. Singh S. (2023). Bacterial volatile organic compounds as biopesticides, growth promoters and plant-defense elicitors: current understanding and future scope. *Biotech. Advan*. 63:108078. 10.1016/j.biotechadv.2022.108078 36513315

[B33] Ryu C. M. Farag M. A. Hu C. H. Reddy M. S. Wei H. X. Kloepper J. W. (2004). Bacterial volatiles induce systemic resistance in *Arabidopsis*. *Plant Physiol*. 134 1017–1026. 14976231 10.1104/pp.103.026583 PMC389924

[B34] Ryu C. M. Farag M. A. Hu C. H. Reddy M. S. Wei H. X. Pare P. W. (2003). Bacterial volatiles promote growth in *Arabidopsis*. *Proc. Natl. Acad. Sci. U.S.A.* 100 4927–4932. 12684534 10.1073/pnas.0730845100 PMC153657

[B35] Scala A. Allmann S. Mirabella R. Haring M. A. Schuurink R. C. (2013). Green leaf volatiles: a plant’s multifunctional weapon against hervibores and pathogens. *Int. J. Mol. Sci.* 14 17781–17811. 23999587 10.3390/ijms140917781 PMC3794753

[B36] Schulz-Bohm K. Gerards S. Hundscheid M. Melenhorst J. de Boer W. Garbeva P. (2018). Calling from distance: attraction of soil bacteria by plant root volatiles. *ISME J*. 12 1252–1262. 29358736 10.1038/s41396-017-0035-3 PMC5931972

[B37] Suárez-Estrella F. Jurado M. M. López-González J. A. Toribio A. Martínez-Gallardo M. R. Estrella-González M. J. (2023). Seed priming by application of *Microbacterium* spp. strains for control of *Botrytis cinerea* and growth promotion of lettuce plants. *Sci. Horticult.* 313:111901.

[B38] Tahir H. A. S. Gu Q. Wu H. Raza W. Hanif A. Wu L. (2017). Plant growth promotion by volatile organic compounds produced by *Bacillus subtilis* SYST2. *Front. Microbiol*. 8:171. 10.3389/fmicb.2017.00171 28223976 PMC5293759

[B39] Tilocca B. Cao A. Migheli Q. (2020). Scent of a killer: microbial volatilome and its role in the biological control of plant pathogens. *Front. Microbiol*. 11:41. 10.3389/fmicb.2020.00041 32117096 PMC7018762

[B40] Tirranen L. S. Gitelson I. I. (2006). The role of volatile metabolites in microbial communities of the LSS higher plant link. *Adv. Space Res.* 38 1227–1232.

[B41] Weise T. Kai M. Piechulla B. (2013). Bacterial ammonia causes significant plant growth inhibition. *PLoS One* 8:e63538. 10.1371/journal.pone.0063538 23691060 PMC3655192

[B42] Weise T. Kai M. Gummesson A. Troeger A. von Reub S. Piepenborn S. (2012). Volatile organic compounds produced by the phytopathogenic bacterium *Xanthomonas campestris* pv. *vesicatoria* 85-10. *Beilstein J. Org. Chem*. 8 579–596. 22563356 10.3762/bjoc.8.65 PMC3343284

[B43] Wheatley R. E. (2002). The consequences of volatile organic compound mediated bacterial and fungal interactions. *Anton. Van Leeuwen.* 81 357–364. 10.1023/a:1020592802234 12448734

[B44] Yaghoubi Khanghahi M. Spagnuolo M. Filannino P. Minervini F. Crecchio C. (2024). Genetic and ecological inheritance of plant growth-promoting rhizobacteria. *Plant Soil.* 10.1007/s11104-024-06852-y

[B45] Zhang H. Sun Y. Xie X. Kim M.-S. Dowd S. E. Pare P. W. (2009). A soil bacterium regulates plant acquisition of iron via deficiency-inducible mechanisms. *Plant J.* 58 568–577. 19154225 10.1111/j.1365-313X.2009.03803.x

[B46] Żuchowska K. Filipiak W. (2023). Modern approaches for detection of volatile organic compounds in metabolic studies focusing on pathogenic bacteria: current state of the art. *J. Pharm. Anal.* 14:100898. 10.1016/j.jpha.2023.11.005 PMC11022102 38634063

